# IUC24402-77 Preliminary results of 177Lu-PSMA-617 priming with enzalutamide in metastatic castration-resistant prostate cancer: a single-arm phase 2 trial (Lu-PRIME)

**DOI:** 10.1093/oncolo/oyaf248.036

**Published:** 2025-08-29

**Authors:** P Aggarwal, S Satapathy, A Sood, S Goyal, G Prakash, B R Mittal

**Affiliations:** Post Graduate Institute of Medical Education and Research, Chandigarh, India; Post Graduate Institute of Medical Education and Research, Chandigarh, India; Post Graduate Institute of Medical Education and Research, Chandigarh, India; Post Graduate Institute of Medical Education and Research, Chandigarh, India; Post Graduate Institute of Medical Education and Research, Chandigarh, India; Post Graduate Institute of Medical Education and Research, Chandigarh, India

## Abstract

**Background:**

Novel antiandrogen therapy (enzalutamide) is integral in treating metastatic castration resistant prostate cancer (mCRPC). Recent preclinical/clinical studies have shown that enzalutamide increases the PSMA expression of PC cells and may enhance the therapeutic efficacy of ^177^Lu-PSMA-617. We aim to evaluate the effect of enzalutamide priming on ^177^Lu-PSMA therapy outcomes in mCRPC patients.

**Methods:**

Histologically proven mCRPC patients (visceral metastases in 5 patients) with prior history of receiving at least one taxane with/without androgen receptor pathway inhibitors, referred for ^177^Lu-PSMA-617 therapy, were prospectively recruited. Short-course enzalutamide (160 mg per day) was given one week before and after ^177^Lu-PSMA-617 therapy. Patients already on enzalutamide discontinued it for 4 weeks to achieve adequate plasma clearance before restarting it according to the priming regimen. ^177^Lu-PSMA-617 was administered intravenously (∼7.4 GBq/cycle at 6-8 weekly intervals, up to 4 cycles). The primary endpoint was the best PSA response rate, and secondary endpoints were objective response rate (ORR), adverse events (AEs) using CTCAE v5, progression-free survival, and overall survival.

**Results:**

Twenty mCRPC patients (median age 67 years [IQR 59-71]) received a total of 50 cycles of ^177^Lu-PSMA-617 (range 1-4) with a median cumulative activity of 15.5 GBq (IQR 14.8-29.5) along with enzalutamide priming. PSA50 response was achieved in 10/20 patients (50%). Based on RECIST 1.1 evaluation in 18 patients, the ORR was 28% (5/18 patients) while the DCR was 39% (7/18 patients). Grade 1/2 AEs were observed in all patients, with fatigue being most common, and anemia the most common hematological AE. Only Grade 3 AEs were observed in 4 patients (20%). Over a median follow-up of 15.6 months (95% CI, 11.9-19.3), 15 events of PSA PD, 15 events of radiological PD and 10 deaths occurred. The median PSA PFS of 4.3 months (95% CI, 3.7-4.9 months), median radiological PFS of 4.6 months (95% CI, 4.4-4.8 months), and median OS of 15.2 months (95% CI, 4-26.3 months) were observed, respectively.

**Conclusions:**

Enzalutamide priming did not significantly improve the disease outcome in mCRPC patients treated with ^177^Lu-PSMA-617 compared to historical data; however, the final outcome is still awaited in this ongoing study. Multicentric randomized control studies are required for validation.

